# Mechanisms of human umbilical cord mesenchymal stem cells-derived exosomal lncRNA GAS5 in alleviating EMT of HPMCs via Wnt/β-catenin signaling pathway

**DOI:** 10.18632/aging.204719

**Published:** 2023-05-23

**Authors:** Yuling Huang, Jianfei Ma, Yi Fan, Lina Yang

**Affiliations:** 1 Department of Geriatrics, The First Hospital of China Medical University, Shenyang, Liaoning 110001, China; 2 Department of Nephrology, The First Hospital of China Medical University, Shenyang, Liaoning 110001, China

**Keywords:** peritoneal dialysis, human umbilical cord mesenchymal stem cell, exosome, human peritoneal mesothelial cell, epithelial-mesenchymal transition

## Abstract

Background: Prolonged peritoneal dialysis (PD) can result in epithelial-to-mesenchymal transition (EMT) and peritoneal fibrosis (PF), which can cause patients to discontinue PD. It is imperative to urgently investigate effective measures to mitigate PF. This study aims to reveal mechanisms of exosomal lncRNA GAS5 derived from human umbilical cord mesenchymal stem cells (hUC-MSCs) on EMT of human peritoneal mesothelial cells (HPMCs) under high glucose (HG) conditions.

Methods: HPMCs were stimulated with 2.5% glucose. The effects on EMT of HPMCs were observed by using an hUC-MSC conditioned medium (hUC-MSC-CM) and extracted exosomes. After hUC-MSCs were transfected with GAS5 siRNA, exosomes were extracted to act on HPMCs for detecting EMT markers, PTEN, and Wnt/β-catenin pathway, lncRNA GAS5 and miR-21 expressions in HPMCs.

Results: We found that HG could induce the EMT of HPMCs. Compared with the HG group, the hUC-MSC-CM could alleviate the EMT of HPMCs induced by HG through exosomes. Exosomes in the hUC-MSC-CM entered HPMCs, by transferring lncRNA GAS5 to HPMCs, which down-regulates miR-21 and up-regulates PTEN, thus finally alleviating EMT of HPMCs. The Wnt/β-catenin pathway plays an essential role in alleviating EMT of HPMCs by exosomes in the hUC-MSC-CM. By transferring lncRNA GAS5 to HPMCs, exosomes derived from hUC-MSCs may competitively bind to miR-21 to regulate suppression on target PTEN genes and alleviate EMT of HPMCs through the Wnt/β-catenin pathway.

Conclusions: Exosomes from the hUC-MSCs-CM could alleviate the EMT of HPMCs induced by HG via regulating lncRNA GAS5/miR-21/PTEN through the Wnt/β-catenin signaling pathway.

## INTRODUCTION

Peritoneal dialysis (PD) is one of the important alternative therapies for end-stage renal diseases (ESRD), however, owing to the peritonea of long-term PD patients being repeatedly exposed to high-glucose (HG) dialysate, biologically incompatible dialysate can make peritoneal mesothelial cells (PMCs) lose their normal morphologies and functions, eventually leading to ultrafiltration failure (UFF) and peritoneal fibrosis (PF) [1]. In recent years, studies show that epithelial-mesenchymal transition (EMT) of human peritoneal mesothelial cells (HPMCs) induced by HG was an initiating and reversible step of PF in PD patients [2]. Therefore, exploring the mechanisms of occurrence and effective measures to delay, or even reverse, the progression of EMT has important theoretical and clinical significance for prolonging the dialysis time and improving the quality of life of ESRD patients.

In recent years, stem cells have received extensive attention and clinical and basic experiments on stem cell therapy have been widely reported. Stem cell therapy has proven to be able to improve multi-system fibrosis including blood, heart, liver, and kidney [3–6]. Due to advantages, such as more powerful proliferation and differentiation ability, convenient access to materials, and low immunogenicity, human umbilical cord mesenchymal stem cells (hUC-MSCs) have better prospects for application compared with other stem cells [7]. At present, the application of MSCs in the treatment of various systemic diseases has become a focus for researchers, but its mechanisms remain to be explored. In contrast to previous theory of differentiation, paracrine gets an increasing attention to explain why MSCs could achieve therapeutic purposes in many disease models [8].

Exosomes, as important paracrine substances of MSCs, contain a variety of proteins, non-coding ribonucleic acids (RNAs), deoxyribonucleic acids (DNAs), and messenger RNAs (mRNAs), serve as important carriers for information transmission among cells [9]. Recent studies by other researchers found that exosomes derived from hUC-MSCs could alleviate fibrosis of organs, such as the liver, lung, and kidney [10–12]. The latest research reports that exosomes in PD effluents could cause peritoneal injury by transferring substances, such as proteins, thus causing the EMT of PMCs [13].

The latest studies indicate that there was a specific expression profile of long non-coding RNA (lncRNA) in fibrous tissues, such as the lung, kidney, liver, and heart: these differentially expressed lncRNAs may play an important regulatory role in the occurrence and development of fibrosis [14]. lncRNA was found to also exist in exosomes [15], accounting for about 20% of exosomes in some patients [16], while the role of lncRNA in exosomes in regulating the EMT of PMCs has rarely been reported. A study showed that downregulation of lncRNA growth-arrest-specific transcript 5 (lncRNA GAS5) expression in serum exosomes could be utilized to identify patients with early-stage NSCLC [17]. Additionally, lncRNA GAS5 enriched in MSC-derived exosomes could alleviate chronic recalcitrant wound healing by regulating inflammatory pathways [18].

Previous studies conducted by this team show that miR-21 targeting phosphatase and tensin homolog deleted on chromosome ten (PTEN) plays an important role during the EMT of HPMCs [19]. Furthermore, some studies indicate that lncRNA GAS5 could be used as competing endogenous RNA (ceRNA) to competitively bind to miR-21, to regulate the suppression of miR-21 on target PTEN genes, thus regulating EMT of tumor cells [20].

Recent studies showed that the Wnt signaling pathway plays an important role in organ fibrosis and EMT, and they could be classified into canonical and non-canonical pathways according to different modes of action. Wnt/β-catenin canonical signaling pathway has been proven to be involved in the occurrence of fibrosis of the myocardium, kidney, and liver [21–23]. Furthermore, PTEN was found to be able to regulate EMT by reducing the level of Wnt [24], indicating that Wnt may interact with PTEN to regulate the progression of EMT of HPMCs.

The current study aimed to determine whether hUC-MSCs derived exosomal lncRNA GAS5 could competitively bind to miR-21 to regulate suppression of target PTEN genes and alleviate EMT of HPMCs through the Wnt/β-catenin signaling pathway.

## MATERIALS AND METHODS

### Cell culture and transfection

Human peritoneal mesothelial cell line, HPMCs was established by Professor Pierre Ronco of French Tenon Hospital [25] and donated by Professor Xu Huimian and Doctor Nadi of The First Hospital of China Medical University. HPMCs were cultured in a culture dish with a diameter of 60 mm with Dulbecco’s modified Eagle medium (DMEM, Gibco) and then cultured in an incubator in an atmosphere containing 5% CO_2_ at 37°C by adding 10% fetal bovine serum (Serapro). Fresh complete culture medium was replaced every 2d. When HPMCs were cultured to complete confluence, they were passaged and grouped. HPMCs were cultured with 2.5% HG for 48 h and were then collected for subsequent testing. lncRNA GAS5 siRNA and negative control (NC) were purchased from GenePharma (Shanghai, China). According to the instructions provided by the manufacturers, GAS5 siRNA and NC were transfected with Lipofectamine 3000 (Invitrogen). The transfection efficiency was verified by real-time polymerase chain reaction (PCR) analysis within 24 h after transfection.

hUC-MSCs and stem cell culture medium were purchased from Shenyang Engineering Technology R&D Center of Cell Therapy Co., Ltd. (Liaoning Province, China). When cells were cultured to about 80% confluence, the culture medium was replaced with fresh serum-free medium, and the hUC-MSC-CM was collected after further culturing for 48 h. hUC-MSC-CM was collected for downstream experiments after being filtered through a 0.22-μm filter. Characterization of MSC plastic adhesion is observed under a light microscope. Flow analysis revealed MSC-specific surface markers, CD73, CD90, and CD14 (eBioscience, USA). Osteogenesis, adipogenesis, and chondrogenesis were confirmed by Alizarin Red, Oil Red, and Alcian Blue staining (Cyagen, China).

293 T cells were cultured in a 24-well plate with Dulbecco's modified Eagle medium (DMEM, Gibco) and then cultured in an incubator in an atmosphere containing 5% CO_2_ at 37°C by adding 10% fetal bovine serum (Serapro).

### Relevant experiments on exosomes

#### Extraction and identification of exosomes

The cultural hUC-MSC-CM was pre-filtered through a 0.22-μm PVDF filter (Millipore, USA), and the exosomes were collected by ultracentrifugation at 100,000 g for 2 h at 4°C followed by classical methods reported in the literature [26]. The morphologies of extracted exosomes were observed by negative staining through an electron microscope (NOVEL). Moreover, Zeta view PMX110 (Particle Metrix) equipped with the Nanoparticle Tracking Analysis (NTA) software (ZetaView 8.02.28) was used for size determination and quantitative analysis of exosomes.

#### Exosome labeling

Those HPMCs in the logarithmic growth phase were inoculated on a 24-well plate at a seeding density of 10^4^/mL, with 1 mL in each well. After being cultured overnight under 5% CO_2_ at 37°C, the cell confluence reached about 20%. The cells were washed three times with PBS (phosphate-buffered saline), and cultured in the replaced serum-free medium under 5% CO_2_ at 37°C. Those exosomes labeled as PKH67 were added. After 6 h of culture, the uptake of exosomes by cells was observed. By utilizing an inverted fluorescence microscope, the uptake of exosomes by cells were observed and photographed under irradiation by light (485 nm) after 6 h.

Nuclear staining: the fixed cells were incubated with 4% PFA for 15 min at room temperature and then incubated with 5 μg/mL of 4′,6-diamidino-2 phenylindole (DAPI) for 10 min under protection against exposure to light at room temperature. The cells were washed with PBS. Moreover, by using an inverted fluorescence microscope, nuclear staining was observed and photographed under irradiation by light (365 nm) using a 40× objective lens.

### Western blot detection

HPMCs were collected in each group and fully lysed with radioimmunoprecipitation assay for 15 to 20 min. The cells were centrifuged for 15 min at 12,000 rpm at a low temperature and the protein concentration was quantified by the bicinchoninic acid (BCA) method. The samples of 40 ug were subjected to 10% sodium dodecyl sulfate-polyacrylamide gel electrophoresis (SDS-PAGE) (Beyotime) and electro-transferred onto polyvinylidene fluoride (PVDF) film for 120 min at a constant current of 200 mA. Then, the samples were sealed for 1 to 2 h at room temperature in 5% non-fat dry milk/Tris-buffered saline Tween (TBST). The primary antibodies (rabbit monoclonal α-SMA, rabbit monoclonal E-cadherin, rabbit monoclonal Vimentin, rabbit monoclonal PTEN, rabbit monoclonal Wnt3a, rabbit monoclonal β-catenin, rabbit monoclonal CD63, rabbit monoclonal TSG101, and mouse monoclonal β-actin antibodies were obtained from Abcam) were diluted with TBST, and the corresponding bands were incubated overnight from primary antibody dilution buffer at 4°C. The secondary antibodies were incubated for 1 to 2 h at room temperature and bands were developed by the electrochemiluminescence (ECL) method. Experiments were repeated at least 3 times.

### Real-time PCR detection

The total RNAs of cells were extracted according to instructions provided with the Trizol kit and complementary DNAs (cDNAs) were synthesized by reverse transcription (RT) according to the instructions provided with the TAKARA reverse transcription kit. The primer sequences of real-time PCR were in Table 1. The reaction system included upstream and downstream primers of separately 1.0 ul, SYBR Premix Ex TapII of 12.5 ul, cDNA template of 2.0 ul, and ddH_2_O of 8.5 ul (the reaction system volume was 25 ul in total). The reaction conditions were as follows: pre-denaturation at 95°C for 30 s, denaturation at 95°C for 5 s, and annealing at 60°C for 30 s (for a total of 45 cycles). The relative expression of mRNAs was expressed as 2^−ΔΔCt^ × 100%.

**Table 1:** The primer sequences of real-time PCR.

**Target**	**Primer sequences**
lncRNA GAS5	Forward primer: 5′-CAGATGCAGTGTGGCTCTGGA-3′
	Reverse primer: 5′-TGTGTGCCAATGGCTTGAGTTAG-3′
β-catenin	Forward primer: 5′-CCTTCCTGGGCATGGAGTC-3′
	Reverse primer: 5′-GAGGAGCAATGATCTTGATCTTC-3′
Wnt3a	Forward primer: 5′-GCCATCGGTGACTTCCTCAA-3′
	Reverse primer: 5′-TTGAAGTAGGTGTAGCGCGG-3′
E-cadherin	Forward primer: 5′-GTCACTGACACCAACGATAATCCT-3′
	Reverse primer: 5′-TTTCAGTGTGGTGATTACGACGTTA-3′
α-SMA	Forward primer: 5′-CCTCCCTTGAGAAGAGTTACGA-3′
	Reverse primer: 5′-GATGCTGTTGTAGGTGGTTTCA-3′
Vimentin	Forward primer: 5′-CAGTCACTCACCTGCGAAGT-3′
	Reverse primer: 5′-AGTTAGCAGCTTCAAGGGCA-3′
GAPDH	Forward primer: 5′-GCACCGTCAAGGCTGAGAAC-3′
	Reverse primer: 5′-TGGTGAACACGCCAGTGGA-3′
miR-21-5p	RT primer: 5′-GTCGTATCCAGTGCAGGGTCCGAGGTATTCGCACTGGATACGACTCAACATCAGT-3′
	Forward primer: 5′-GGCGGTAGCTTATCAGACTGATG-3′
	Reverse primer: 5′-GTGCAGGGTCCGAGGTATTC-3′
U6	RT primer: 5′-AACGCTTCACGAATTTGCGT-3′
	Forward primer: 5′-CTCGCTTCGGCAGCACA-3′
	Reverse primer: 5′-AACGCTTCACGAATTTGCGT-3′

### Immunofluorescence

HPMCs were grown to 60~70% confluence following which they were cultured with HG or exosomes for 48 h. Cells were fixed with 4% paraformaldehyde for 15 min after being washed with PBS 3 times, then blocked in 5% Bovine Serum Albumin V for 1 h at 37°C. The primary antibodies (rabbit monoclonal β-catenin obtained from Abcam) were diluted with 1% Bovine Serum Albumin V, and 6-well plates were incubated overnight from primary antibody dilution buffer at 4°C. The secondary antibodies (TRITC Goat Anti-Rabbit IgG from Earthox) were incubated for 1 h at 37°C protected from light and the nuclei were stained by DAPI. Images were taken with an inverted fluorescence microscope.

### Dual-luciferase reporter gene assay

PmirGLO Dual-Luciferase miR Target Expression Vector (Promega) was used to verify the targeting relationship between lncRNA GAS5 and miR-21. The wild-type reporter construct mirGLO-GAS5 or the mutant reporter construct pmirGLO-GAS-mut (miR-21) was co-transfected with miR-21 mimic or miR-Control in 293 T cells. Then we verified the targeting relationship between PTEN and miR-21. The wild-type reporter construct pmirGLO-PTEN or the mutant reporter construct pmirGLO-PTEN-mut (miR-21) was co-transfected with miR-21 mimic or miR-Control in 293 T cells. After transfection for 48 h, the luciferase activity of each group of cells was detected using a Dual-Luciferase Reporter Assay System (Promega). Each experiment was repeated at least three times.

### Statistical analysis

All data were statistically processed using SPSS21.0 software. The measurement data were expressed as mean ± SEM and one-way analysis of variance (ANOVA) was used for comparison between groups; *p* < 0.05 represented a difference of statistical significance.

### Availability of data and material

The data used to support the findings of this study are available from the corresponding author upon request.

## RESULTS

### Identification of hUC-MSCs

**Figure 1:**
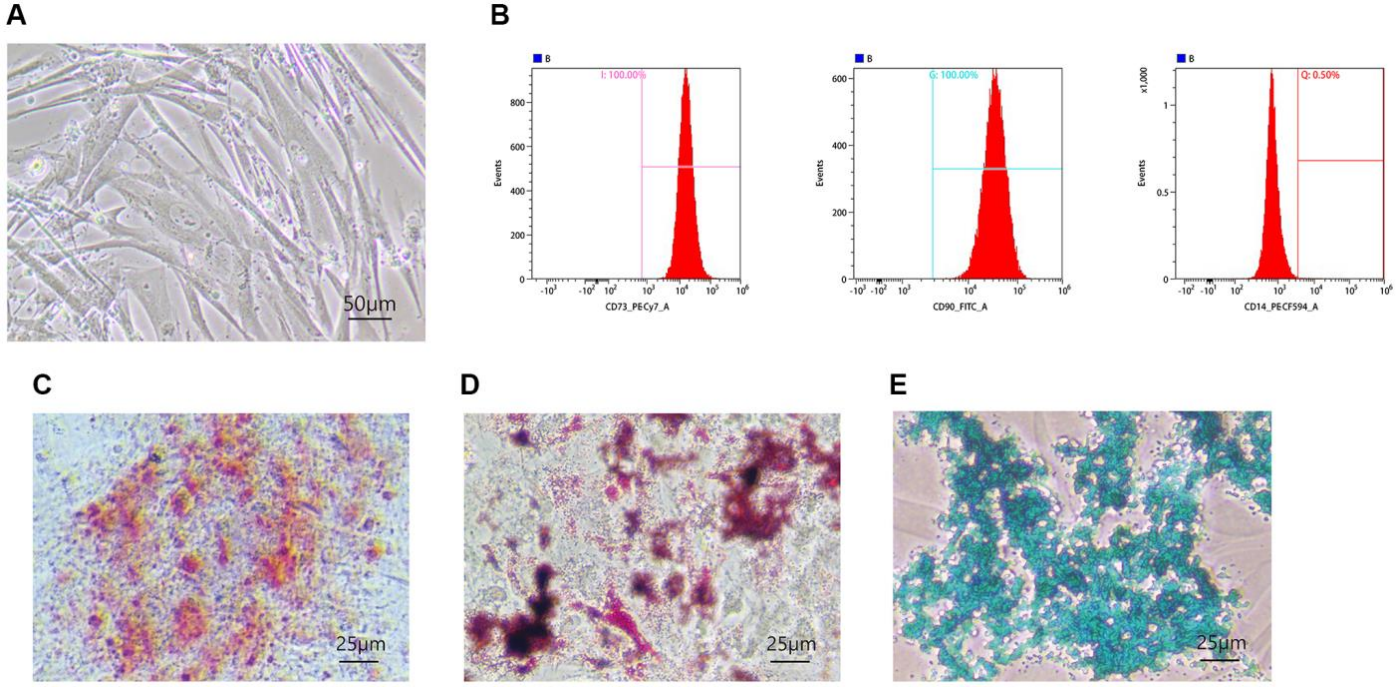
**Identification of hUC-MSCs.** (**A**) The morphologies of hUC-MSCs were observed under a light microscope. (**B**) Flow analysis revealed MSC specific surface markers, CD73, CD90, and CD14. (**C**–**E**) Osteogenesis (**C**), adipogenesis (**D**), and chondrogenesis (**E**) were confirmed by Alizarin Red, Oil Red, and Alcian Blue staining.

According to ISCT (the International Society for Cellular Therapy), three criteria are proposed to define MSCs: adherence to plastic, specific surface antigen expression, and multipotent differentiation potential [27]. The 4–5th generation hUC-MSCs adhered to the wall and grew in a fibroblast-like shape under a light microscope (Figure 1A). A flow analysis revealed that the surface of hUC-MSCs was positively stained with CD73 (100%), CD90 (100%), and negatively with CD14 (0.50%) (Figure 1B). 21 days after osteogenesis induction, brownish red opaque calcium nodules appeared (Figure 1C). Oil Red O staining of numerous lipid droplets indicated adipogenic differentiation (Figure 1D). The endogenous acid mucopolysaccharide in cartilage tissue by Alcian Blue staining proved the chondrogenic differentiation ability of HUMSCs (Figure 1E). The above results indicate that hUC-MSCs have been successfully identified.

### Identification of exosomes

**Figure 2:**
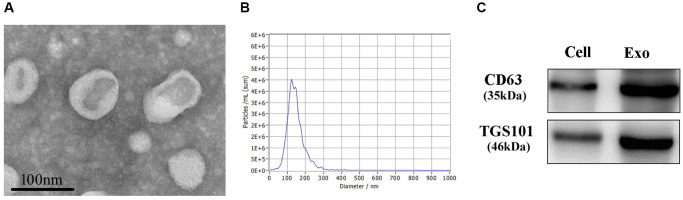
**Identification of exosomes.** (**A**) The morphologies of exosomes were observed through an electron microscope. (**B**) NTA was used for the size determination of exosomes. (**C**) Western Blot was used to measure CD63 and TSG101 proteins.

Electron microscopic results showed that exosomes in the saucer-like structures could be clearly observed in the samples of the hUC-MSC-CM (Figure 2A). NTA results indicated that the average size of exosome vesicles was 135.7 nm (Figure 2B). Western Blot assay results suggested that CD63 and TSG101 proteins were significantly expressed (Figure 2C). These results demonstrated that exosomes had been successfully extracted from the hUC-MSC-CM.

### hUC-MSC-CM alleviating EMT of HPMCs stimulated by HG through exosomes

**Figure 3:**
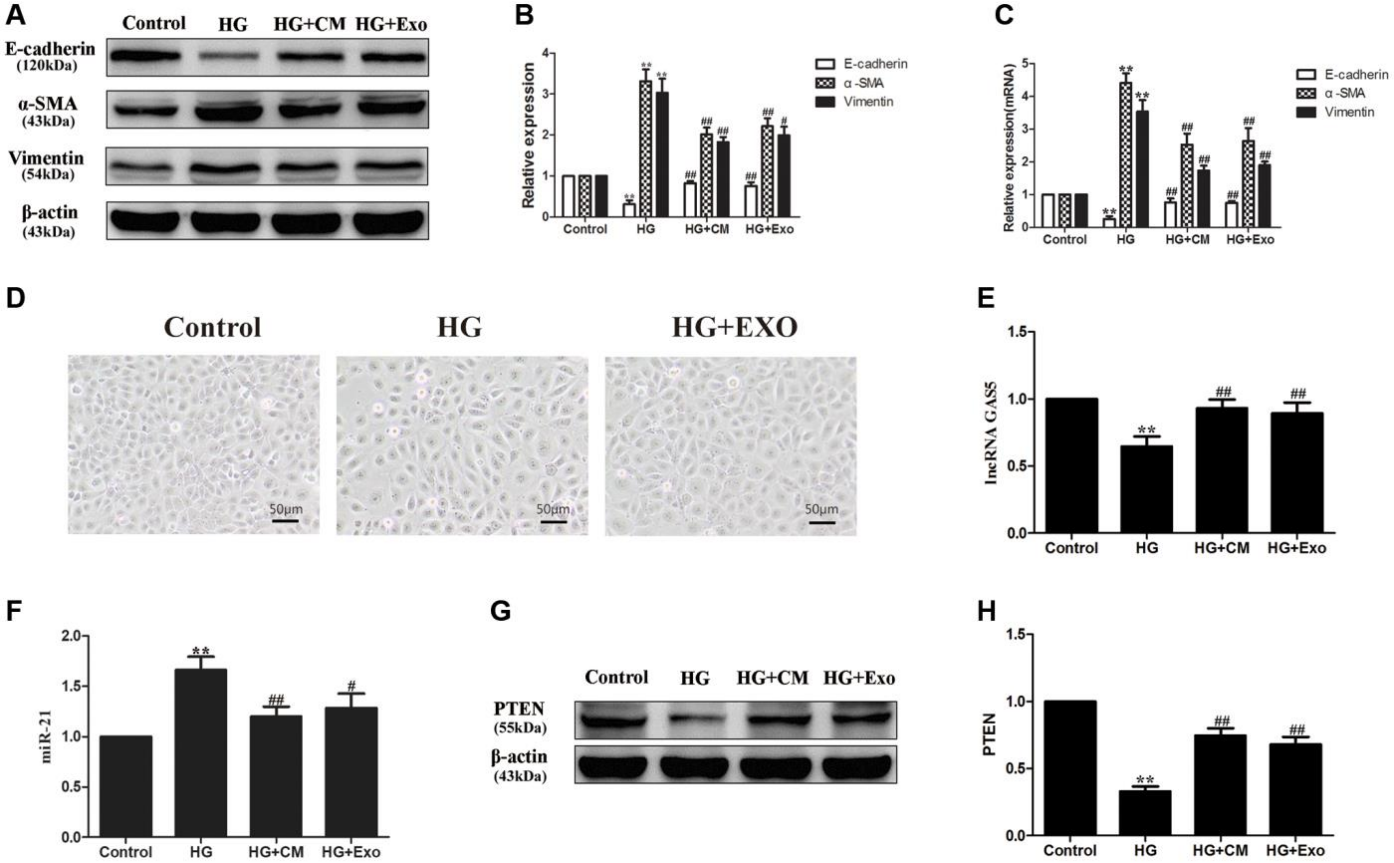
**hUC-MSCs-CM alleviating EMT of HPMCs stimulated by HG through exosomes.** (**A**) HG stimulation induced EMT of HPMCs, the hUC-MSC-CM mainly alleviated EMT through exosomes by Western Blot. (**B**) Statistical results of EMT changes after HG and hUC-MSCs/hUC-MSCs exosomes treatment. (**C**) The expression of EMT markers was detected by real-time PCR in HPMCs. (**D**) Treatment with hUC-MSCs exosomes effect cell morphology in HG-treated HPMCs. (**E**) The expression of lncRNA GAS5 was detected by real-time PCR. (**F**) The expression of miR-21 was detected by real-time PCR. (**G**, **H**) The expression of PTEN was detected by Western Blot. Each value represents the mean ± SEM (*n* = 3) (^**^P < 0.01 vs. Control, ^##^*P* < 0.01 vs. HG, ^#^*P* < 0.05 vs. HG).

Compared with the control group, HPMCs stimulated by 2.5% HG significantly up-regulated expressions of α-SMA and vimentin and down-regulate the expression of E-cadherin, indicating that HG stimulation induced EMT of HPMCs (Figure 3A–3C). In comparison with the HG stimulation group, the hUC-MSC-CM could alleviate the EMT of HPMCs induced by HG, as do exosomes extracted from the hUC-MSC-CM, suggesting that the hUC-MSC-CM mainly alleviates EMT through exosomes. Compared with the control group, the number of HPMCs decreased and showed a shuttle shape after adding 2.5% HG for 48 hours. In contrast to the HG group, light microscopy shows a decrease in shuttle cells and an increase in the number of cells after exosome treatment (Figure 3D).

Furthermore, it was found that HG treatment could down-regulate the expression of lncRNA GAS5 in HPMCs and the treatments with the hUC-MSC-CM and with exosomes restored the expression of lncRNA GAS5 (Figure 3E). According to previous results, lncRNA GAS5 can competitively combine with miR-21 to regulate PTEN and influences EMT in HPMCs [28]. Here, we endeavored to explore the effects of hUC-MSC-CM and exosomes on the expressions of miR-21 and target PTEN in the HG-induced HPMCs. Expressions of miR-21 and target PTEN proteins were also detected and the results showed that the expression of miR-21 was up-regulated (Figure 3F), while that of PTEN was down-regulated (Figure 3G, 3H) in HPMCs stimulated by HG, the hUC-MSC-CM and exosomes down-regulated the expression of miR-21 (Figure 3F) and restored the expression of PTEN (Figure 3G, 3H). Based on the above results, it was considered that the hUC-MSCs-CM transfer lncRNA GAS5 through exosomes, thus alleviating EMT of HPMCs stimulated by HG through lncRNA GAS5/miR-21/PTEN.

### Detection of exosomes entering recipient cells

**Figure 4:**
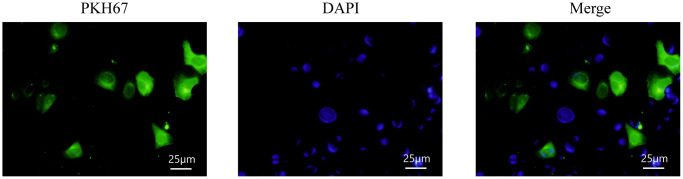
Detection of exosomes entering recipient cells.

Exosomes derived from hUC-MSCs were labeled with PKH67 and finally used to incubate HPMCs. It was found that PKH67 was expressed in HPMCs incubated with exosomes, indicating that exosomes secreted by hUC-MSCs could enter recipient cells, namely HPMCs (Figure 4).

### Exosomal lncRNA GAS5 from hUC-MSCs competitively bind to miR-21 to regulate suppression on target PTEN genes to alleviate EMT of HPMCs

**Figure 5:**
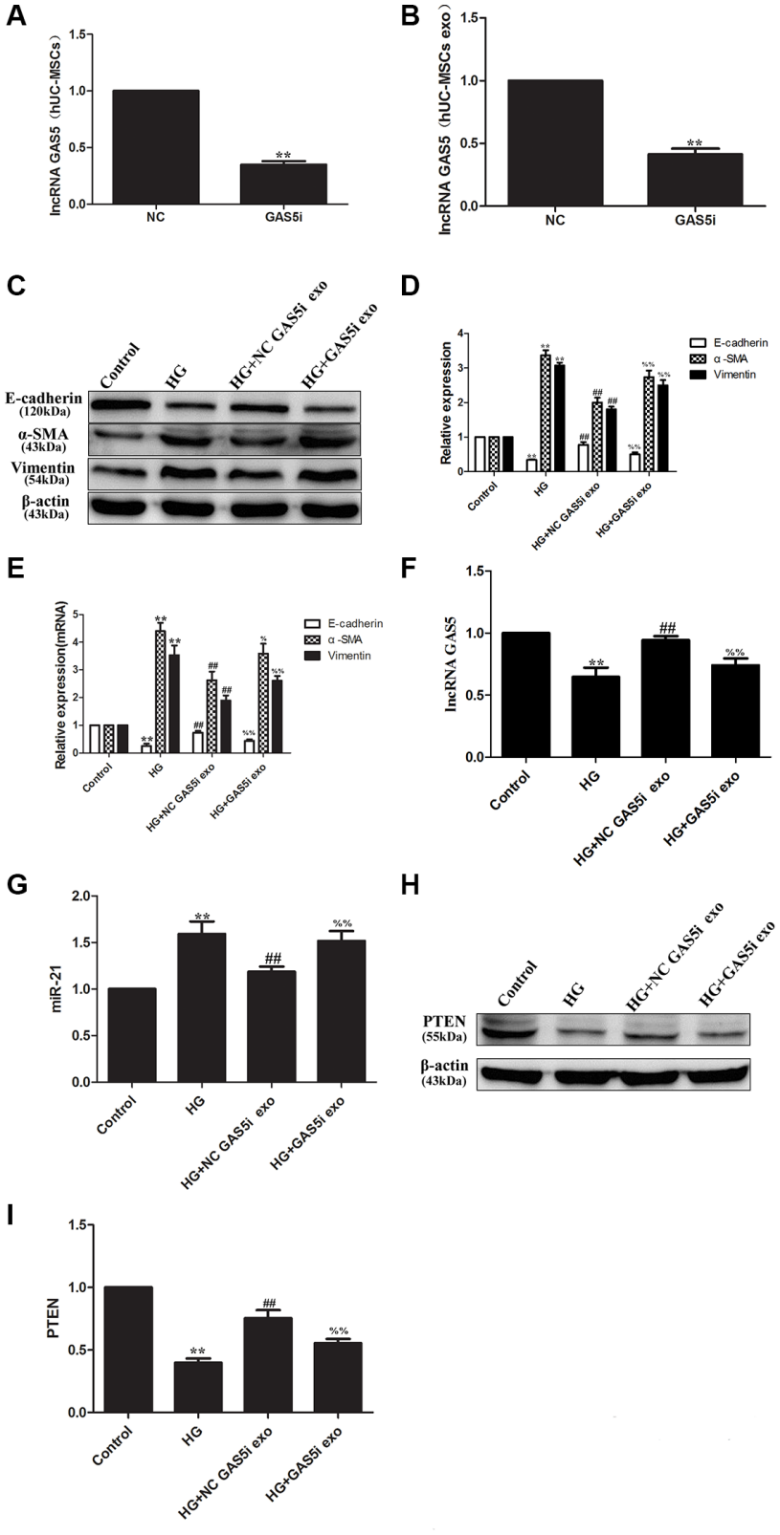
**Exosomal lncRNA GAS5 from hUC-MSCs regulate suppression on target PTEN genes to alleviate EMT of HPMCs.** (**A**, **B**) hUC-MSCs were transfected with lncRNA GAS5 siRNA, and the expression of lncRNA GAS5 was detected by real-time PCR in hUC-MSCs (**A**) and hUC-MSCs exosomes (**B**). (**C**) The expression of EMT markers was detected by Western Blot in HPMCs. (**D**) Statistical results of EMT changes after HG and hUC-MSCs/hUC-MSCs exosomes treatment. (**E**) The expression of EMT markers was detected by real-time PCR in HPMCs. (**F**) The expression of lncRNA GAS5 was detected by real-time PCR in HPMCs. (**G**) The expression of miRNA-21 was detected by real-time PCR in HPMCs. (**H**) The expression of PTEN was detected by Western Blot in HPMCs. (**I**) Statistical results of PTEN changes after HG and hUC-MSCs/hUC-MSCs exosomes treatment. Each value represents the mean ± SEM (*n* = 3) (^**^P < 0.01 vs. Control, ^##^*P* < 0.01 vs. HG, ^%%^*P* < 0.01 vs. HG+ NC GAS5i exo, ^%^*P* < 0.05 vs. HG+ NC GAS5i exo).

To verify that exosomal lncRNA GAS5 from the conditioned media of hUC-MSCs regulate EMT of HPMCs, hUC-MSCs were transfected with GAS5siRNA, so that the expression of lncRNA GAS5 was significantly down-regulated in hUC-MSCs and exosomes (Figure 5A, 5B). Compared with the HG group, exosomes secreted from hUC-MSCs NC GAS5i group significantly reduced the levels of α-SMA and vimentin and increased the level of E-cadherin in HG treated HPMCs, indicating that exosomes secreted from hUC-MSCs NC GAS5i could attenuate EMT of HPMCs. Compared with HG+ NC GAS5i exo group, exosomes secreted from hUC-MSCs low-expressing lncRNA GAS5 significantly increased the levels of α-SMA and vimentin and reduced the level of E-cadherin (Figure 5C–5E). Meanwhile, the expression of lncRNA GAS5 in HPMCs was down-regulated in (Figure 5F), the expression of miR-21 was up-regulated (Figure 5G), and the expression of PTEN was significantly down-regulated (Figure 5H, 5I). These data show that exosomal lncRNA GAS5 from hUC-MSCs down-regulate miR-21 and up-regulate PTEN to alleviate EMT of HPMCs.

**Figure 6:**
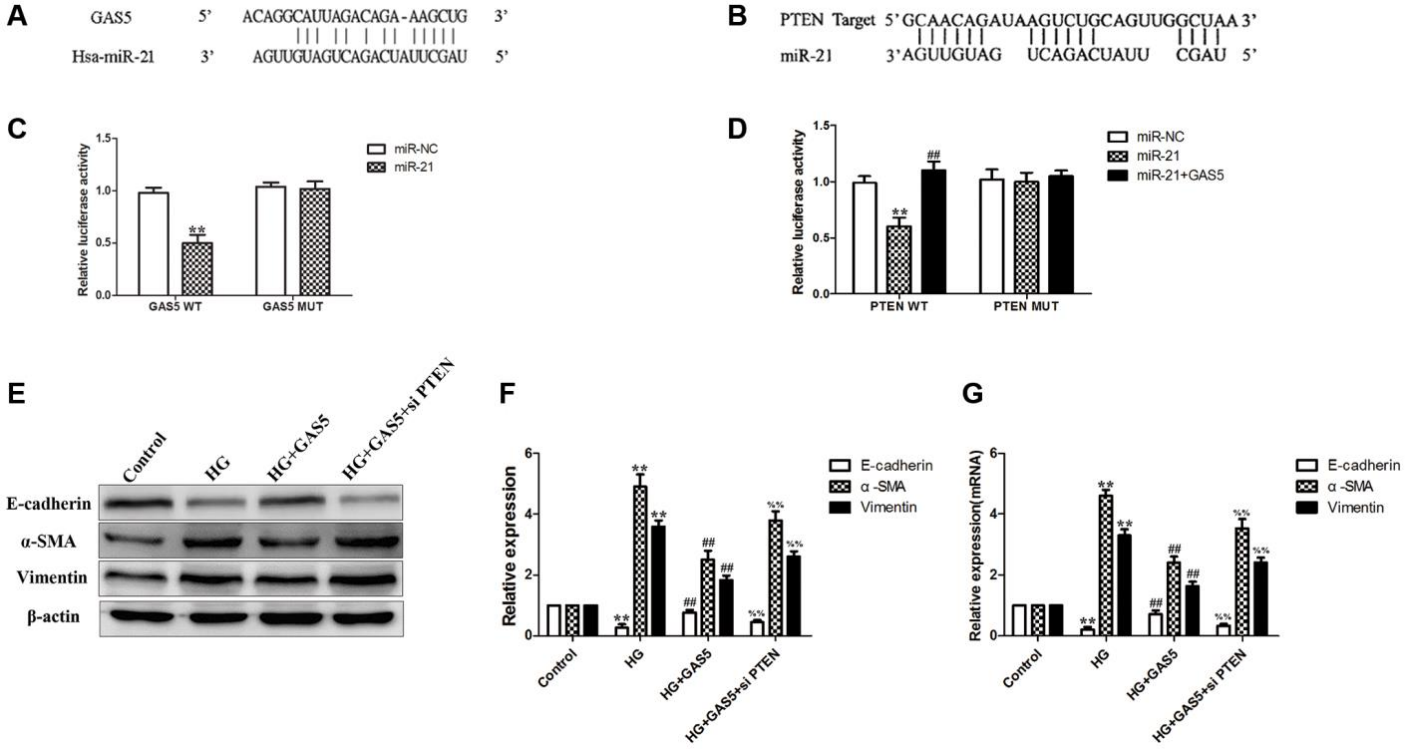
**Exosomal lncRNA GAS5 from hUC-MSCs competitively bind to miR-21 to regulate suppression on target PTEN genes to alleviate EMT of HPMCs.** (**A**) Bioinformatics analysis result showed that lncRNA GAS5 had a binding site with miR-21. (**B**) Bioinformatics analysis result showed that PTEN had a binding site with miR-21. (**C**) Dual-luciferase reporter gene assay was used to analyze the relationship between lncRNA GAS5 and miR-21. (**D**) Dual-luciferase reporter gene assay was used to analyze the relationship among PTEN, miR-21, and lncRNA GAS5. (**E**) Transfection with lncRNA GAS5 significantly alleviated the HG-induced EMT, however, combination with silencing of PTEN reversed the mitigating effect of lncRNA GAS5 on EMT by Western Blot. (**F**) Statistical results of EMT changes after transfection with lncRNA GAS5 in combination with silencing of PTEN. (**G**) The expression of EMT markers was detected by real-time PCR in HPMCs. Each value represents the mean ± SEM (*n* = 3) (**C**, **D**) ^**^P < 0.01 vs. Control, ^##^*P* < 0.01 vs. miR-21; (**E**–**G**) ^**^P < 0.01 vs. Control, ^##^*P* < 0.01 vs. HG, ^%%^*P* < 0.01 vs. HG+ GAS5).

The preliminary study conducted by the research team demonstrated that miR-21 targeting PTEN played an important role in the EMT of HPMCs [19]. According to bioinformatics analysis, the bioinformatics software predicted that the binding sites of miR-21 were contained in lncRNA GAS5 and PTEN (Figure 6A, 6B). Dual-luciferase reporter gene assay was used to verify the interaction between lncRNA GAS5 and miR-21, the relative luciferase activity was significantly decreased in GAS-WT and miR-21 mimic co-transfection group, and luciferase activity was not suppressed in GAS-MUT and miR-21 mimic co-transfection group (Figure 6C). Dual-luciferase reporter gene assay was then used to verify the interaction between PTEN and miR-21, the relative luciferase activity was significantly decreased in PTEN-WT and miR-21 mimic co-transfection group, and luciferase activity was not suppressed in PTEN-MUT and miR-21 mimic co-transfection group. Furthermore, neither in PTEN-WT and miR-21 mimic co-transfection group, nor in PTEN-MUT and miR-21 mimic co-transfection group, transfection with lncRNA GAS5 could suppress the relative luciferase activity (Figure 6D), suggesting that lncRNA GAS5 may act as ceRNA to bind competitively to miR-21, thus regulating the expression of target PTEN genes. Besides, compared to the HG group, transfection with lncRNA GAS5 significantly alleviated the EMT. However, combination with silencing of PTEN reversed the mitigating effect of lncRNA GAS5 on EMT (Figure 6E–6G). These data show that, by transferring lncRNA GAS5 to HPMCs, exosomal lncRNA GAS5 from hUC-MSCs competitively bind to miR-21 to regulate suppression on target PTEN genes, to finally alleviate EMT of HPMCs.

### lncRNA GAS5 in exosomes from hUC-MSCs-CM alleviating EMT through Wnt/β-catenin pathway

**Figure 7:**
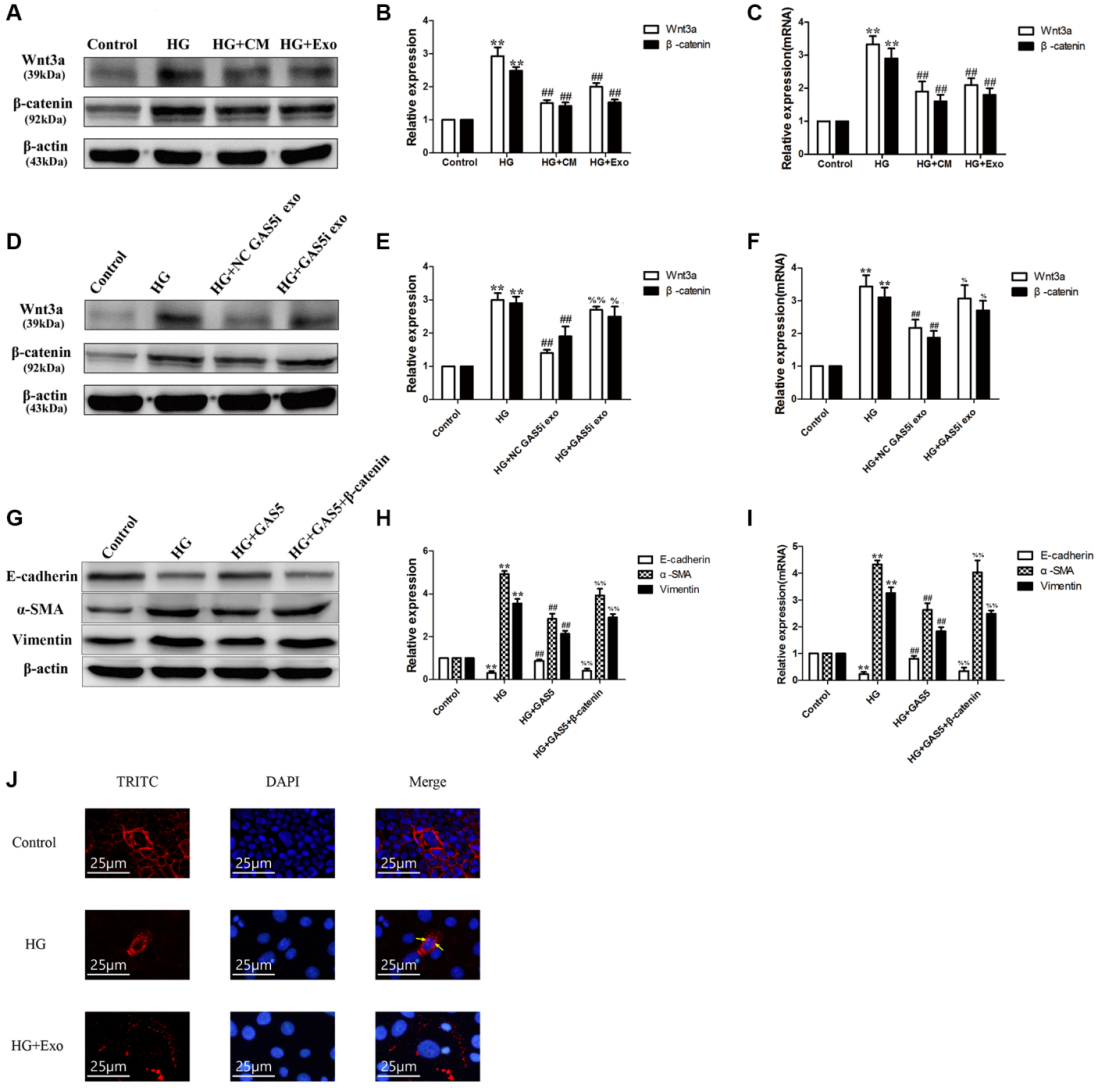
**lncRNA GAS5 in exosomes from hUC-MSCs-CM alleviating EMT through Wnt/β-catenin pathway.** (**A**) Exosomes from hUC-MSCs could suppress the Wnt/β-catenin pathway in HG HPMCs by Western Blot. (**B**) Statistical results of Wnt/β-catenin pathway changes after HG and hUC-MSCs/hUC-MSCs exosomes treatment. (**C**) The expression of Wnt3a and β-catenin was detected by real-time PCR. (**D**–**F**) Exosomes from hUC-MSCs could suppress the Wnt/β-catenin pathway in HG HPMCs by transferring lncRNA GAS5 to HPMCs. (**G**) After transfection with β-catenin vector, alleviated EMT all reversed in HG+ lncRNA GAS5 group by Western Blot. (**H**) Statistical results of EMT changes after HG and transfection with lncRNA GAS5 and β-catenin vector. (**I**) The expression of EMT markers was detected by real-time PCR in HPMCs. (**J**) Exosomes secreted from hUC-MSCs alleviated the transfer into the nucleus of β-catenin in HG-treated HPMCs. (**A**–**F**) ^**^P < 0.01 vs. Control, ^##^*P* < 0.01 vs. HG, ^%%^*P* < 0.01 vs. HG+ NC GAS5i exo, ^%^*P* < 0.05 vs. HG+ NC GAS5i exo; (**G**–**I**) ^**^P < 0.01 vs. Control, ^##^*P* < 0.01 vs. HG, ^%%^*P* < 0.01 vs. HG+ GAS5).

Compared with the control group, HPMCs stimulated by 2.5% HG significantly up-regulated the expressions of Wnt3a and β-catenin proteins and mRNA, indicating that HG could stimulate EMT of HPMCs through the Wnt/β-catenin pathway (Figure 7A–7C). Compared with the HG stimulation group, the hUC-MSC-CM and exosomes could alleviate activation of the Wnt/β-catenin pathway, showing that exosomes from the hUC-MSC-CM could alleviate EMT of HPMCs through Wnt/β-catenin pathway (Figure 7A–7C). Compared with the HG group, exosomes secreted from the hUC-MSCs NC GAS5i group significantly reduced the levels of Wnt3a and β-catenin in HG-treated HPMCs, indicating that exosomes secreted from hUC-MSCs NC GAS5i could attenuate activation of Wnt/β-catenin in HPMCs EMT. Compared with HG+ NC GAS5i exo group, exosomes secreted from hUC-MSCs low-expressing lncRNA GAS5 significantly increased the expressions of Wnt3a and β-catenin (Figure 7D–7F).

To further verify the relationship between lncRNA GAS5 and β-catenin, we transfected HG-induced HPMC with lncRNA GAS5 and β-catenin vector. After transfection with lncRNA GAS5, increased α-SMA, and vimentin levels and decreased E-cadherin expression all reversed in HG group. Compared to HG+ lncRNA GAS5 group, transfection of a activated β-catenin vector attenuated the lncRNA GAS5-mediated protein levels of E-cadherin, α-SMA, and vimentin (Figure 7G–7I). Additionally, transferring into the nucleus of β-catenin in HG-treated HPMCs was alleviated by exosomes secreted from hUC-MSCs (Figure 7J). In a previous study, we also reported that transfection with lncRNA GAS5 regulate β-catenin sub-cellular localization [28]. These results imply that exosomes from the hUC-MSCs-CM could suppress the Wnt/β-catenin pathway to alleviate the EMT of HPMCs by transferring lncRNA GAS5 to HPMCs.

## DISCUSSION

In comparison with traditional hemodialysis, PD had the advantages of simple operation, good removal of meso-molecular substances, slow decline of residual renal functions, and stable hemodynamics, thus saving medical resources and health expenditure to a great extent [29]. However, long-term PD could result in UFF and PF, thus withdrawing from PD. At present, there are emerging effective and feasible methods with which to solve PF. Masola et al. concluded that some strategies to retard PF, including low GDPs and neutral Ph, glucose sparing, use of metabolically active osmolytes, addition of pharmacological agents conventional PD solutions, and target to glycolytic and pyruvate metabolism [30]. However, complex molecular pathways of PF entail exploring the mechanisms of PF and effective measures to alleviate PF in prolonging the survival of PD patients.

EMT of HPMCs was considered as the core step in PF [29]. When EMT occurred in the presence of inflammation or pro-fibrosis stimulation, the phenotypic expression of epithelial cells, such as E-cadherin in HPMCs was suppressed, leading to a loss of cell junction. Furthermore, the cells have fibroblast-like morphologies and the phenotypic expression of mesenchymal cells, such as vimentin and α-SMA in cells was up-regulated, so that the cells could obtain the properties and functions of mesenchymal cells [29]. Finally, this resulted in PF and UUF. This research shows that HG stimulation could induce the EMT of HPMCs.

The therapeutic effects of MSCs on fibrosis of tissues, such as the liver, lung, and kidney have been widely studied and such effects mainly depend on paracrine function [3–6, 8]. As important paracrine substances of MSCs, exosomes contained various proteins, non-coding RNAs, DNAs, and mRNAs, and were important carriers for information transmission among cells [9]. Zhong et al. reported that micro-vesicles derived from mesenchymal stem cells could suppress cell cycle inhibitors P15 and P19 *in vivo* and *in vitro* and restart cell cycles, thus reversing EMT [31]. Grange et al. considered that extracellular vesicles (EVs) derived from human liver stem-like cells (HLSCs) and MSCs could suppress and reverse the progression of fibrosis in a mouse model for diabetic nephropathy. This was realized by down-regulating fibrosis-related gene Serpina1a, FAS ligand, CCL3, TIMP1, MMP3, Collagen I, and Snail [32]. Jin et al. proved that exosomes derived from adipose-derived mesenchymal stem cells (ADMSC-Exo) attenuated the EMT of podocytes by suppressing ZEB2 gene transcription with miR-215-5p [33]. Our research results confirmed that the hUC-MSCs-CM alleviates the EMT of HPMCs through exosomes.

**Figure 8:**
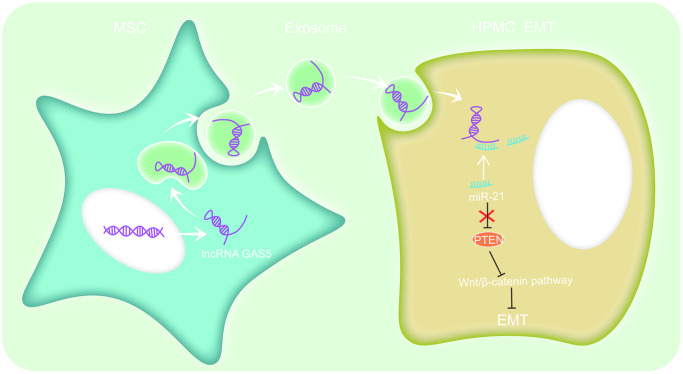
**hUC-MSCs exosomes could alleviate HG-induced EMT in HPMCs.** The mechanism involves regulating lncRNA GAS5/miR-21/PTEN via the Wnt/catenin signaling pathway to alleviate HPMC EMT.

As carriers, exosomes can encapsulate various information molecules, including lipids, proteins, RNA, etc. [34]. Li C et al. suggested that LncRNA GAS5 in exosomes may candidate as an ideal noninvasive marker to identify early NSCLC [17]. LncRNA GAS5 contained in exosomes derived from ADMSCs can modulate inflammation [18]. Additionally, lncRNA GAS5 has been proven to inhibit EMT in prostate cancer [35]. Cesana et al. revealed that lncRNA can be used as ceRNA sponge miRNAs to regulate the expression of downstream genes [36]. As a competing endogenous RNA for miR-96-5p, lncRNA GAS5 promotes renal tubular epithelial fibrosis [37]. Likewise, lncRNA GAS5 directly binds to miR-21 to suppress EMT in human uveal melanoma [38]. By coincidence, our previous research confirmed that lncRNA GAS5 can competitively combine with miR-21 to regulate PTEN and influences EMT in HPMCs [28]. This research further demonstrates that exosomal lncRNA GAS5 from hUC-MSCs competitively binds to miR-21 to regulate suppression on target PTEN genes to alleviate EMT of HPMCs (Figure 8).

An increasing number of studies suggested that the Wnt/β-catenin signaling pathway may be involved in the EMT process of tissues, such as the lung, liver, and kidney [21–23]. When the pathway was activated, Wnt ligands were likely to bind to receptors to activate β-catenin as a nuclear shuttle and bind to T-cell factor/lymphoid enhancer factor (TCF/LEF) to activate the transcription of fibronectin, MMP-7, PAI-1, and target genes, such as Twist and Snail [39]. As shown in the latest studies, by detecting HPMCs in PD effluents of PD patients, it was found that compared with the group undergoing PD for less than one month, the expressions of Wnt and β-catenin in the group undergoing PD for more than one year were significantly up-regulated. Moreover, the detection results of indices including E-cadherin and α-SMA indicated the occurrence of EMT. In addition, *in vitro* and animal experiments also confirm that HG may promote the EMT of HPMCs by activating the Wnt/β-catenin signaling pathway [40–42]. This indicates that the Wnt/β-catenin signaling pathway may be involved in the EMT of peritoneal tissues. The research results also illustrated that the Wnt/β-catenin signaling pathway could be activated after treatment with HG and exosomes from the hUC-MSCs-CM suppress the Wnt/β-catenin pathway by transferring lncRNA GAS5 to HPMCs, thus finally alleviating the occurrence of EMT of HPMCs (Figure 8).

## CONCLUSIONS

In conclusion, exosomes from the hUC-MSCs-CM could alleviate the EMT of HPMCs induced by HG. The mechanism is to regulate lncRNA GAS5/miR-21/PTEN through the Wnt/β-catenin signaling pathway, and ultimately alleviate the occurrence of HPMCs EMT. This study provides a new scientific basis for illustrating the effects and mechanisms of action of exosomes derived from hUC-MSC in PD-related PF and offers a new opportunity to prolong survival time and improve the quality of life of PD patients.
